# Minimizing the makespan and carbon emissions in the green flexible job shop scheduling problem with learning effects

**DOI:** 10.1038/s41598-023-33615-z

**Published:** 2023-04-19

**Authors:** Zhi Li, Yingjian Chen

**Affiliations:** School of Economics and Management, Tiangong University, Tianjin, 300387 China

**Keywords:** Engineering, Mathematics and computing

## Abstract

One of the most difficult challenges for modern manufacturing is reducing carbon emissions. This paper focuses on the green scheduling problem in a flexible job shop system, taking into account energy consumption and worker learning effects. With the objective of simultaneously minimizing the makespan and total carbon emissions, the green flexible job shop scheduling problem (GFJSP) is formulated as a mixed integer linear multiobjective optimization model. Then, the improved multiobjective sparrow search algorithm (IMOSSA) is developed to find the optimal solution. Finally, we conduct computational experiments, including a comparison between IMOSSA and the nondominated sorting genetic algorithm II (NSGA-II), Jaya and the mixed integer linear programming (MILP) solver of CPLEX. The results demonstrate that IMOSSA has high precision, good convergence and excellent performance in solving the GFJSP in low-carbon manufacturing systems.

## Introduction

The concentration of CO_2_ in the Earth’s atmosphere is currently at its highest point in at least 2 million years, and the global warming caused by carbon emissions from human activities cannot be ignored^1^. Carbon emissions from manufacturing industries account for a significant portion of the world’s total carbon emissions. Thus, achieving green manufacturing and reducing carbon emissions are critical to accomplishing the goal of carbon peaking and carbon neutrality^2^. Efficient production scheduling arrangements can effectively reduce resource consumption and environmental pollution in machine production, achieving the goal of energy savings and emission reduction^3^. Therefore, it is of great significance to achieve green manufacturing in production scheduling.

In production scheduling, the flexible job shop scheduling problem (FJSP) considers the flexibility of scheduling, where multiple different machines can process the same operations. It breaks the restriction of unique production resources and is more suitable to actual production situations^4,5^. In green manufacturing environments, the green flexible flow-shop scheduling problem (GFJSP) is an extension of the FJSP in which environmental criteria such as energy consumption, carbon emissions, and noise pollution are captured^6,7^. In the literature, the GFJSP is a research hotspot and has received more attention in recent years. Foumani & Smith-Miles^8^ studied an energy-efficient job shop scheduling problem and proposed a series of mixed-integer linear programming models to reduce the carbon emissions of machines during processing. Li et al.^9^ studied a multiobjective FJSP model considering machine loading, the maximum completion time of all jobs, and total carbon emissions and designed an improved artificial bee colony algorithm (IABC) to solve the model. Gong et al.^10^ studied the GFJSP with laborer flexibility, considering total completion time and energy consumption as the two objective functions, and proposed a solution method based on an evolutionary algorithm and variable neighborhood search (VNS). Afsar et al.^11^ proposed a fuzzy job shop scheduling problem considering non-processing energy minimization and used e a new enhanced memetic algorithm to obtain a feasible solution. In 2022, Shao et al.^12^ examined an energy-efficient distributed flexible-flow shop scheduling problem. They proposed an energy-saving strategy to reduce the machine speed for noncritical work without changing the makespan. Later, García Gómez et al.^13^ focused on minimizing energy consumption for the FJSP and developed a memetic algorithm that combines the global search with local search for model solving. Current studies on job shop scheduling problems assume that the processing time from the first job to the last one is known and fixed. However, in real manufacturing processes, workers can become more efficient over time, and the processing time is correspondingly reduced, which causes a ‘learning effect’^14^. Wright^15^ first discovered the learning effect in the aircraft industry. Since then, it has been applied in many industries. Biskup^16^ introduced this concept into machine scheduling problems and solved a single-machine scheduling problem with polynomials. In recent years, the scheduling problem considering the learning effect has attracted increasing attention from scholars. Jiang et al.^17^ studied *seru* scheduling problems considering past-sequence-dependent setup times and DeJong’s learning effect. It was shown that relaxing the assumption of the learning coefficients can reduce the complexity of the problem. In Jemmali and Hidri^18^, the parallel machine scheduling problem with a learning effect and with maximum lateness as the objective function was studied. In this work, a genetic algorithm and other heuristics were developed for the studied scheduling problem. Later, Zhang et al.^19^ focused on the cell scheduling problem with Dejong’s learning effect consideration in an assembly line production system, where a logic-based benders decomposition method was developed to solve the problem. Although much research has been conducted to study the effect of learning in various industries, research on the learning effect in the job shop scheduling problem is relatively sparse. Current studies mainly focus on the economic concerns of the scheduling strategy, such as the problems of minimizing the makespan and total flow time with learning effects in traditional job shop scheduling. Unfortunately, few studies consider learning effects as a constraint in the GFJSP with environmental concerns. The consideration of learning effects in the GFJSP is necessary because studies have shown that with improved worker learning effects, the processing time can be reduced to two-thirds of the original time^20^, which ultimately leads to a reduction in both makespan and carbon emissions.

It is well known that the job shop scheduling problem is strongly NP-hard^21^. An intelligent optimization algorithm can efficiently solve this problem, achieve the reasonable allocation of resources, and improve production efficiency. The sparrow search algorithm (SSA) was first proposed by Xue and Shen^22^. It is a novel intelligent optimization algorithm inspired by the behavior of sparrow populations. Compared with particle swarm optimization (PSO), the gray wolf optimizer (GWO), lightning attachment procedure optimization (LAPO) and other learning algorithms, SSA has better solving ability and higher efficiency for complex global optimization problems^23^. Zhang and Ding^24^ proposed chaotic SSA with logistic mapping to optimize a stochastic configuration network (SCN) in solving massive-scale data problems. Zhu & Yousefi^25^ employed an adaptive learning factor to refine SSA and used adaptive SSA to identify the parameters of the optimal model for proton exchange membrane fuel cell (PEMFC) reactors. Tian and Chen^26^ designed an improved sparrow search algorithm by introducing Cauchy variation and reverse learning for ultrashort-term wind speed prediction. Wu et al.^27^ proposed a fast stochastic configuration network (FSCN) and adopted the adaptive adjusting hyperparameter and mutation strategy to improve SSA. Dong et al.^28^ showed that using niche optimization technology and Levy flights to carry out chaotic transformation in SSA improved the optimization ability.

Existing research on SSA shows that the algorithm performs well in solving NP-hard problems, but no research has yet applied it to the FJSP. In particular, the GFJSP considering energy consumption is a typical discrete optimization problem (the job processing time is often a discrete variable in the GFSP). In this study, to make SSA available for discrete problems, an improved SSA is developed to minimize the makespan as well as the total carbon emissions. Experiments are performed to verify the effectiveness of the improved algorithm and to expand the application scenarios of SSA.

In summary, considering the great pressure on the environment caused by fossil energy, reducing carbon emissions in job shops is not only environmentally attractive but also economically beneficial for manufacturers^29^. As seen from the reports in the literature, most studies only consider energy consumption as an optimization objective of the FJSP, while few have considered the factor of worker efficiency. In many realistic settings, workers can improve their efficiency continuously over time. Thus, the learning effect significantly influences the practicability of the final scheduling scheme. There is, however, little work on the FJSP considering worker learning effects. From a novel perspective, this paper indicates that the study of the multiobjective low-carbon-emission GFJSP with learning effect constraints has extremely high theoretical value and practical meaning. There are at least three contributions of the current study:

- A novel low-carbon scheduling problem for a flexible job shop environment, named green flexible job shop scheduling problem (GFJSP) with a constrained learning effect, is investigated.
- A mixed-integer programming (MIP) model is developed with the objective of simultaneously minimizing the makespan and total carbon emissions. In the MIP model, carbon emissions are generated by the machines during job processing as well as by idle machines.
- An improved multiobjective sparrow search algorithm (IMOSSA) is proposed. The strategies proposed in IMOSSA can greatly improve computational efficiency and overall quality. Therefore, the effectiveness of IMOSSA for solving GFJSP is verified.

The remainder of this paper is organized as follows: the multiobjective optimization model is formulated in Section “Model formulation”, including a detailed description of the green flexible job shop scheduling problem considering the learning effect. Then, a mixed integer linear multiobjective optimization model is formulated in Section “Improved multiobjetive sparrow search algorithm (IMOSSA)”, and the solution method IMOSSA is proposed. In Section “Computational experiments”, computational experiments are carried out, and the results are reported and analyzed. Finally, Section “Conclusion” presents conclusions and further research opportunities.

## Model formulation

### Problem description

A GFJSP considering worker learning effects can be stated as follows: *n* jobs will be processed on *m* machines. The *i*th ($1 \le i \le n$) job is composed of *n*_*i*_ operations, and each operation can be processed by multiple machines. Each operation generates different amounts of carbon emissions. The machines are started at instant 0 and stopped after the last operation is completed. For this problem, two objectives are chosen to reflect both production and energy efficiency: the makespan (*C*_*max*_) and total carbon emissions (*TCE*). Concretely, the makespan is defined as the time it takes to complete the last job on machine $k \, \left( {1 \le k \le m} \right)$. In actual production, a small amount of carbon emissions are generated when machines are kept idle. Thus, we assume that there are two types of carbon emissions: one is generated by machines during job processing, and the other is generated by idle machines due to energy consumption.

The machine processing time changes under the influence of the learning effect. The learning effect causes the service time of a given task to be determined by its position in the processing sequence. That is, if a job is scheduled for a later time, its processing time will be shorter. With the increasing number of the same type of job being processed, workers become more experienced, and the processing time decreases. The actual processing time changes according to Dejong’s learning effect curve^30^. The change in the actual processing time will affect the carbon emissions generated by the machine and thus further affect the accuracy of the scheduling model. Based on the characteristics of batch production in flexible job shops^31^, Dejong’s learning effect model^32^ is used to solve the FJSP. The job processing time is:

1 $$ P_{ijk} = \overline{P}_{ijk} \left[ {M + (1 - M)r^{\alpha } } \right], $$

where $P_{ijk}$ represents the processing time of operation *O*_*ij*_ on machine* k*, $\overline{P}_{ijk}$ is the standard processing time of operation *O*_*ij*_ on machine* k*, *r* is the processing order of operation *O*_*ij*_ on machine* k*, *M* is the incompressibility factor $0 \le M \le 1$, and $\alpha$ is the learning index, where $\alpha \le 0$.

### Notation

The notation of the GFJSP is listed in Table 1.

**Table 1:** Main symbols and descriptions.

Symbol	Description
*n*	The number of jobs
*m*	The number of machines
*n*_*i*_	The number of operations for job *i*
*i, l*	The index of jobs, *i*, *l* = 1, 2,…, *n*
*j, p*	The index of operations, *j* = 1, 2,…, *n*_*i*_ ; *p* = 1, 2,…, *n*_*i*_
*k*	The index of machines, *k* = 1, 2,…, *m*
*O*_*ij*_	The *j*th operation of job *i*
$L_{ijk} \, =$	$$\left\{ \begin{gathered} 1,{\text{ if operation }}O_{ij} {\text{ is processed on machine }}k{;} \hfill \\ 0,{\text{ otherwise}}{.} \hfill \\ \end{gathered} \right.$$
$L^{\prime}_{ijklp} \, =$	$$\left\{ \begin{gathered} 1,{\text{ if operation }}O_{ij} {\text{ is the precedence of operation }}O_{lp} , \, O_{ij} {\text{ and }}O_{lp} {\text{ are processed on machine }}k \, ; \hfill \\ 0,{\text{ otherwise}}. \hfill \\ \end{gathered} \right.$$
*S*_*ijk*_	The starting time of operation *O*_*ij*_ on machine *k*
*P*_*ijk*_	The processing time of operation *O*_*ij*_ on machine *k*
*C*_*ijk*_	The ending time of operation *O*_*ij*_ on machine *k*
*C*_*max*_	The makespan, which is the completion time of the last operation on the last machine
*E*_*ijk*_	The carbon emissions for operation *O*_*ij*_ on machine *k*, *E*_*ijk*_ > 0
*RC*_*k*_	The carbon emissions per unit time when machine *k* is idle, *RC*_*k*_ > 0
*R*_*k*_	The carbon emissions when machine *k* is idle,* R*_*k*_ > 0
*TCE*	The total carbon emissions

### Assumptions

The multiobjective optimization model of the GFJSP is formulated under the following assumptions:

1. At any time, each worker can only process on one machine, and the worker cannot leave the machine during processing.
2. The learning effect of a worker is only related to the number of operations he or she has processed on the machine;
3. The worker and the machine are considered an operating unit, and the machine number is used to indicate this unit.
4. When an operation has begun to be processed, it should be completed without any interruption;
5. Each machine can only process one operation at each moment, and an operation can only be processed by an available machine.

Assumptions (1) ~ (3) are based on Chen, Wu and Lee^33^; assumption (1) guarantees the continuity of the learning effect on the same machine. Assumption (2) emphasizes the correlation between the learning effect and the number of operations processed by the machine. Assumption (3) is based on assumption (1); since the learning effect of a worker is related to the machine he or she operates, the presentation is simplified by treating the worker and the machine as an operating unit.

### Modeling

The objective of the GFJSP with the learning effect considered in this study is to minimize the makespan and carbon emissions. Based on this, a mixed integer linear multiobjective optimization model is established as follows.

2 $$ f = \min \left( {C_{\max } ,TCE} \right), $$

3 $$ C_{\max } = \mathop {\max }\limits_{i,j,k} L_{ijk} C_{ijk} , $$

4 $$ TCE = \sum\limits_{k = 1}^{m} {\sum\limits_{j = 1}^{{n_{i} }} {\sum\limits_{i = 1}^{n} {L_{ijk} E_{ijk} } } } + \sum\limits_{k = 1}^{m} {R_{k} } . $$

Subject to

5 $$ R_{k} = RC_{k} \times \left( {\mathop {\max }\limits_{i,j} L_{ijk} C_{ijk} - \sum\limits_{j = 1}^{{n_{i} }} {\sum\limits_{i = 1}^{n} {L_{ijk} P_{ijk} } } } \right), $$

6 $$ C_{ijk} \le S_{i(j + 1)k} , $$

7 $$ S_{ijk} + P_{ijk} \le S_{lpk} + (1 - L^{\prime}_{ijklp} )Q, $$

8 $$ \sum\limits_{k = 1}^{m} {L_{ijk} } = 1, $$

9 $$ S_{ijk} + L_{ijk} P_{ijk} = C_{ijk} , $$

10 $$ S_{ijk} \ge 0, $$

11 $$ C_{ijk} \ge 0. $$

In the model above, Eq. (2) is the multiobjective function considering the learning effect. Equation (3) is the makespan objective, and Eq. (4) is the total carbon emissions objective. Equation (5) represents the carbon emissions of machine *k* when it is idle. Equation (6) indicates the precedence relation. That is, there are restrictions on the processing sequence between the operations of the same job. Equation (7) means that each machine can only process one job at a time, where *Q* is a very large positive number. Equation (8) is the decision variable that restricts an operation to be processed by only one available machine. Equation (9) states that after starting, the process cannot be interrupted. Equations (10) and (11) indicate that the starting time and ending time of each operation should be positive.

## Improved multiobjetive sparrow search algorithm (IMOSSA)

### Sparrow search algorithm (SSA)

SSA is a novel global optimization algorithm that has the advantages of a strong searching ability, fast convergence and robustness in high-dimensional problems^24^. Therefore, SSA is adopted to solve the proposed multiobjetive GFJSP. In SSA, there are two types of populations: producers and scroungers. Individual fitness values and searching space to find food sources are higher among the producers. The scroungers search for food based on the producer who can provide the best food. As soon as a sparrow sees danger, it gives a timely warning signal, and the whole population immediately adopts antipredation behavior. The positions of the sparrow populations are updated through foraging and antipredation behavior.

SSA is a competitive algorithm in which the individual (producer) with a higher cost value has a higher chance of finding food in the solution space. The specific implementation mechanism of SSA is as follows:

1. To update the food area, the producers search a wide area and constantly update their locations. The update strategies of the producers are given in Eq. (14).
2. The scroungers follow the producers to forage for food to obtain better fitness values. The update strategies of the scroungers are given in Eq. (15).
3. As the threat of predators persists, 10% ~ 20% of sparrows will be randomly selected as reporters for monitoring to alert the whole population to engage in antipredation behavior when predators appear. The update strategies of the reporters are given in Eq. (16).

### Modified sparrow search algorithm applied to the GFJSP

**Figure 1:**
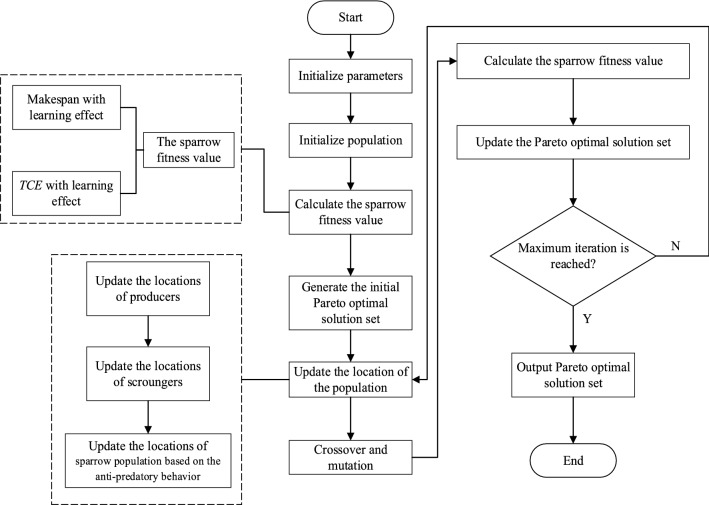
IMOSSA procedure for minimizing the makespan and *TCE* in the GFJSP with the learning effect.

The GFJSP is a typical discrete optimization problem. To make SSA available for discrete problems, we design a two-segment coding scheme. Additionally, to boost SSA’s global optimization ability and promote population variety, we introduce the position-based crossover (PBX) operator^34^ and the exchange mutation operator in the algorithm. Moreover, for the simultaneous optimization of the makespan and the *TCE*, we construct the Pareto optimal solution set. For basic information on multiobjective optimization concepts such as the Pareto optimal solution set and domination, one can refer to Tamssaouet et al.^35^. In each iteration, the sparrows look for workable answers, discover nondominant solutions, and include them in the Pareto optimal solution set. In addition, old solutions that are dominated by the new ones will be deleted from the Pareto optimal solution set. The procedure of IMOSSA is depicted in Fig. 1, and the following are the steps involved.

Step 1: Initialize the parameters to generate the first sparrow population.

Step 2: Calculate the fitness values of makespan and *TCE* with the learning effect for the first sparrow population and then generate the initial Pareto optimal solution set.

Step 3: Update the positions of the sparrow population.

Step 4: Perform PBX crossover and exchange mutation.

Step 5: Calculate the fitness values of makespan and *TCE* with the learning effect for the new sparrow population.

Step 6: Update the Pareto optimal solution set.

Step 7: If IMOSSA has reached the maximum number of iterations, go to Step 8; otherwise, return to Step 3.

Step 8: Output the Pareto optimal solution set.

### Encoding and decoding

**Figure 2:**
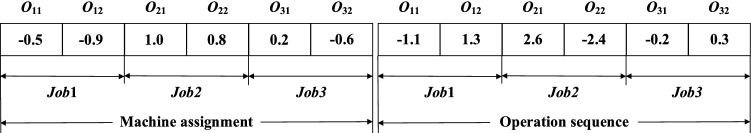
Individual sparrow position encoding scheme.

The encoding strategy is a crucial stage of IMOSSA implementation. The GFJSP involves two subproblems: machine assignment and operation scheduling. To achieve reasonable scheduling, a two-segment encoding method is proposed according to the characteristics of the GFJSP. That is, the locations of sparrows in IMOSSA consist of two segments: the machine assignment $\left[ {sw_{1} ,sw_{2} ,sw_{3} , \ldots ,sw_{H} } \right]$ and operation sequence $\left[ {sw^{\prime}_{1} ,sw^{\prime}_{2} ,sw^{\prime}_{3} , \ldots ,sw^{\prime}_{H} } \right]$. The dimension of searching space of each segment is *H* ($H = \sum\nolimits_{i = 1}^{n} {n_{i} }$); then, the total dimension of searching space of the sparrows is 2*H*. For example, the location of a sparrow can be defined as $SW = \left[ {sw_{1} ,sw_{2} ,sw_{3} , \ldots ,sw_{H} ;sw^{\prime}_{1} ,sw^{\prime}_{2} ,sw^{\prime}_{3} , \ldots ,sw^{\prime}_{H} } \right]$, where $sw_{h} ,sw^{\prime}_{h} \left( {1 \le h \le H} \right)$ follows a uniform distribution of $[ - \mu , \, \mu ]$. Let $\mu$ be an arbitrary positive integer. Each $sw_{h}$ ($sw^{\prime}_{h}$) represents an operation. As shown in Fig. 2, we assume that there are 3 jobs in the GFJSP, each job has 2 operations, and the value of $\mu$ is 3. The first segment is the machine assignment, and the second segment is the operation sequence.

The conversion mechanism is used to translate between the locations of sparrows and the scheduling schemes. Similar to the two-segment encoding method, the conversion process involves the conversion of machine assignments and of operation sequences.

#### Conversion of machine assignments

On the basis of the conversion method proposed by Yuan, Xu and Yang^36^, the individual sparrow positions are converted into machine indices, as shown in Eq. (12), where $z(h)$ indicates the number of available machines for the operation corresponding to $sw_{h}$. $k(h)$ is the machine allocated to the operation indicated by $sw_{h}$.

12 $$ k(h) = round\left( {\frac{{sw_{h} + \mu }}{2\mu }\left( {z(h) - 1} \right) + 1} \right),{ 1} \le h \le H. $$

#### Conversion of operation sequences

**Figure 3:**
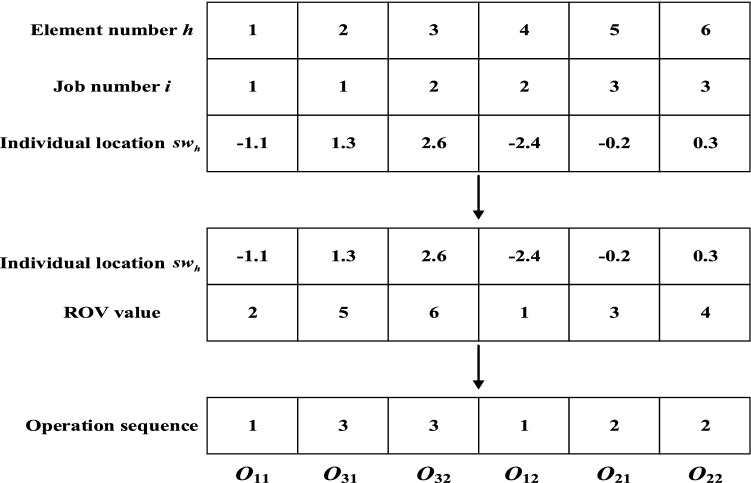
Operation sequence segment conversion.

According to the ranked order value (ROV) rule^37^, the sparrow locations $sw^{\prime}_{h}$ are sorted in ascending order. Each $O_{ij}$ is marked with a unique ROV value, and then the operations can be sorted based on the same ROV value as that of $sw^{\prime}_{h}$. As Fig. 3 shows, each operation $O_{ij}$ is assigned to a machine from left to right until all operations are allocated.

Decoding is the inverse process of encoding. Through decoding, the scheduling scheme can be converted to individual sparrow positions. Similarly, the decoding process is divided into machine assignment segment decoding and operation sequence segment decoding.

#### Machine assignment segment decoding

13 $$ sw_{h} = \frac{2\mu }{{z(h) - 1}}\left( {u(h) - 1} \right) - \mu , \, z(h) \ne 1. $$

Each machine assignment segment is decoded according to Eq. (13). If $z(h) = 1$, let $sw_{h}$ be a random number in the range $[ - \mu , \, \mu ]$.

#### Operation sequence segment decoding

**Figure 4:**
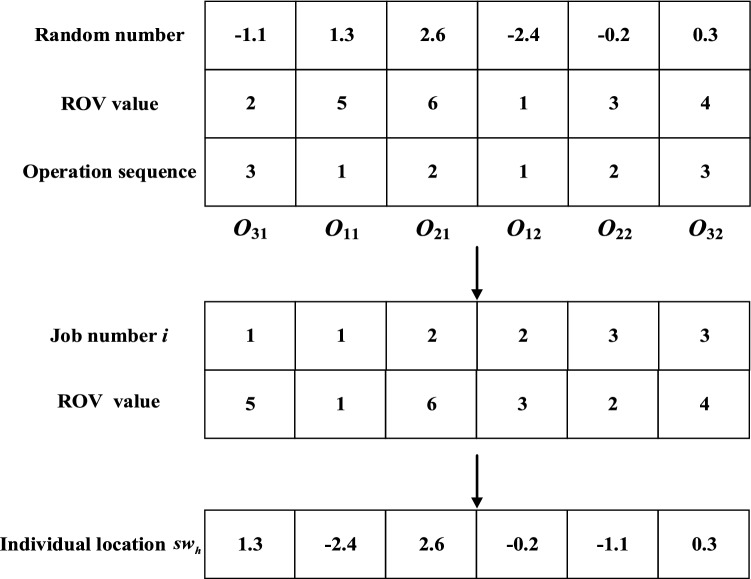
Operation sequence segment decoding.

As shown in Fig. 4, we generate *H* random numbers in the range $[ - \mu , \, \mu ]$ and assign ROV values to them in ascending order. The random numbers and the ROV values are sorted according to the operation sequence.$sw^{\prime}_{h}$ is determined by the ROV value.

### Population initialization and updating

In IMOSSA, each sparrow represents a potential solution. Through foraging behavior and antipredation behavior, the locations of the sparrow population are updated to obtain a higher fitness value.

The locations of producers are updated by Eq. (14):

14 $$ X_{ite}^{t + 1} = \left\{ \begin{gathered} X_{ite}^{t} \cdot \exp \left( { - \frac{ite}{{\theta \cdot iter_{\max } }}} \right){, }\quad if \, V < ST, \hfill \\ X_{ite}^{t} + W \cdot G, \, otherwise. \hfill \\ \end{gathered} \right. $$

Here, *t* refers to the current iteration number.$iter_{\max }$ indicates the maximum iteration number. $X_{ite}^{t}$ represents the location of the *ite*th sparrow. $\theta$ is a random number in the range (0, 1].* W* is a random number from a normal distribution. *G* is a one-dimensional matrix with a value of 1 for each member. *V* ∈ [0, 1] denotes the alert value, and *ST* ∈ [0.5, 1] indicates the safety critical value. When *V* < *ST*, producers are able to conduct large-scale foraging with no surrounding predators. When *V* ≥ *ST*, the sparrow population is in danger and should quickly fly to other safe areas.

The locations of scroungers are updated by Eq. (15):

15 $$ X_{ite}^{t + 1} = \left\{ \begin{gathered} W \cdot \exp \left( {\frac{{X_{worst}^{t} - X_{ite}^{t} }}{{ite^{2} }}} \right), \, if \, ite > pop/2, \hfill \\ X_{b}^{t + 1} + |X_{ite}^{t} - X_{b}^{t + 1} |A^{ + } \cdot G, \, otherwise. \hfill \\ \end{gathered} \right. $$

$X_{b}^{t + 1}$ is the best position at the (*t* + 1)th iteration, and $X_{worst}^{t}$ denotes the worst position at the *t*th iteration. *A* is a $1 \times d$ matrix in which each element is given a value of 1 or -1 at random, and $A^{ + } = A^{T} \left( {AA^{T} } \right)^{ - 1}$. *pop* is the population size. When $ite > pop/2$, it means that the scrounger is unable to obtain food and must go to another location to do so. When $ite \le pop/2$, the scrounger should forage near $X_{b}^{t}$.

The antipredation behavior of the sparrow population is based on Eq. (16):

16 $$ X_{ite}^{t + 1} = \left\{ \begin{gathered} X_{{\text{b}}}^{t} + \beta \cdot |X_{ite}^{t} - X_{b}^{t} |, \, \quad if \, F_{ite} > F_{best} , \hfill \\ X_{ite}^{t} + K \cdot \left( {\frac{{|X_{ite}^{t} - X_{worst}^{t} |}}{{|F_{ite} - F_{w} | + \delta }}} \right){, } \quad if \, F_{ite} = F_{best} . \hfill \\ \end{gathered} \right. $$

where $\beta$ is a random number that follows a standard normal distribution with parameters 0 and 1.* K* is randomly distributed within the range [− 1, 1]. $F_{ite}$ indicates the sparrow's current fitness value.$F_{best}$ is the current best global fitness value.$F_{w}$ is the current worst global fitness value.* δ* is a very small constant to avoid a zero denominator.

### PBX crossover operator and exchange mutation operator

To improve the demographic variety of sparrows, after incorporating the features of GFJSP, we add the PBX crossover and exchange mutation operators in the IMOSSA.

#### PBX crossover operator

**Figure 5:**
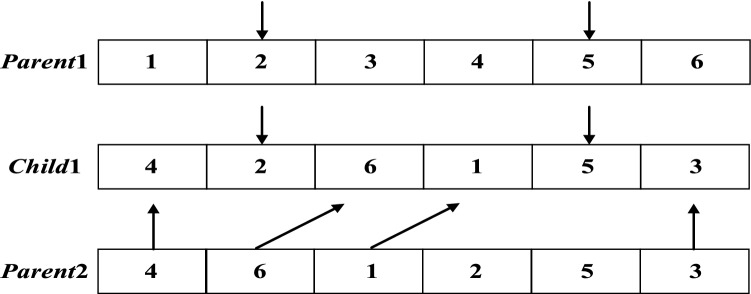
PBX crossover operator.

The process of the PBX crossover operator is shown in Fig. 5. The steps are as follows:

- Step 1: The crossover operation requires two parent sparrows. Randomly select sparrows *Parent*1 and *Parent*2 from the population.
- Step 2: Randomly select elements in several positions in *Parent*1; the positions do not have to be consecutive.
- Step 3: Select the element in *Child*1 that is in the same position as that of *Parent*1.
- Step 4: Find the same element in *Parent*2 as the previously selected element, then insert the other elements of *Parent*2 in the original order in *Child*1.

#### Exchange mutation operator

**Figure 6:**
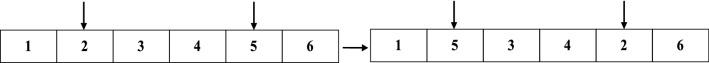
Exchange mutation operator.

Randomly select a sparrow from the population, then select two of its elements and exchange their values, as shown in Fig. 6.

## Computational experiments

In this section, we discuss the computational experiments used to evaluate the performance of the improved algorithm. To validate the efficiency of IMOSSA for solving the GFJSP in Sections “Model formulation” and “Improved multiobjetive sparrow search algorithm (IMOSSA)”, we compare it with validated solution approaches. In previous studies, the nondominated sorting genetic algorithm II (NSGA-II) and Jaya were proven to have good performance in solving multiobjective optimization problems^38,39^, so we chose NSGA-II^40^ and the Jaya algorithm^41^ as the comparison algorithms.

### Experimental settings

The current section compares the performance of the developed solution approaches: IMOSSA, NSGA-II, and Jaya algorithm. To this end, we use job shop scheduling OR-Library data. We consider *n* = 6, 10, 15, 20, 30 and *m* = 5, 6, 10,15, and we generate 11 test cases (3 cases of the FT benchmark and 8 cases of the LA benchmark, where the 11 cases are divided into small, medium and large scale; see Table 2) on the basis of combinations of *n* and *m*. Furthermore, carbon emissions from processing machines and carbon emissions from idle machines are both considered in all cases. To do this, we generate a random test set of carbon emissions. All parameters of the test set and their references are summarized in Tables 3 and 4. All the experiments are performed on a desktop computer with an AMD Ryzen 7 4800H, 2.90 GHz CPU, 16.00 G RAM, Windows 10 64 OS, and MATLAB R2021a.

**Table 2:** Scale division of the test cases.

Problem scale	Test cases ($n \times m$)
Small	FT06 (6 × 6), LA01 (10 × 5), LA06 (15 × 5)
Medium	FT10 (10 × 10), FT20 (20 × 5), LA11 (20 × 5), LA16 (10 × 10)
Large	LA21 (15 × 10), LA26 (20 × 10), LA31 (30 × 10), LA36 (15 × 15)

**Table 3:** Parameters used in the experiments.

Parameter	Parameter level	Reference
Number of jobs (*n*)	6, 10, 15, 20, 30	OR-Library
Number of machines (*m*)	5, 6, 10, 15	OR-Library
Processing time for each operation (*P*_*ijk*_)	Based on 11 classic test cases	OR-Library
Carbon emissions per unit of time with idle machines ($R^{\prime}_{k}$)	Uniform distribution: [1, 10] grams	Ding et al.^4^
Carbon emissions per unit of time with processing machines ($C^{\prime}_{ijk}$)	Uniform distribution: [10, 30] grams	Ding et al.^4^
Incompressibility factor (*M*)	0.5	Sun et al.^42^
Learning index (α)	− 1	Sun et al.^42^

**Table 4:** Parameters used in the metaheuristic algorithms.

Algorithm	Parameter	Parameter level	Reference
IMOSSA	Maximum iterations ($iter_{\max }$)	300	Wu and Sun^3^
	Population size (*pop*)	100	Wu and Sun^3^
	Proportion of producers	0.2, 0.45, 0.7	Tian and Chen^26^
	Proportion of sparrows aware of danger	0.1, 0.2, 0.3	Tian and Chen^26^
	Safety threshold (*ST*)	0.5, 0.65, 0.8	Zhang and Ding^24^
NSGA-II	Maximum iterations ($iter_{\max }$)	300	Tan et al.^40^
	Population size (*pop*)	100	Tan et al.^40^
	Crossover probability	0.5, 0.55, 0.6	Tan et al.^40^
	Mutation probability	0.1, 0.15, 0.2	Tan et al.^40^
Jaya	Maximum iterations ($iter_{\max }$)	300	Rao and Saroj^43^
	Population size (*pop*)	100	Rao and Saroj^43^

### Algorithm parameter setting

The parameter configuration can impact the performance of the algorithm in solving the GFJSP. The proposed IMOSSA contains three important parameters. The proportion of producers *PP*, the proportion of sparrows aware of danger *PD*, and the safety threshold *ST*. The Taguchi approach of Design of Experiment (DOE)^44^ is used at different scales to obtain the optimal combinations of parameters. Each parameter has three levels, *PP* ∈ {0.5, 0.7, 0.8}, *PD* ∈ {0.1, 0.2, 0.3} and *ST* ∈ {0.5, 0.65, 0.8}. Additionally, to reduce the quantity of experiments, Taguchi’s orthogonal experiment method is adopted.

Because three parameters, *PP*, *PD*, and *ST,* need to be tested, according to Taguchi’s orthogonal array L9(3^3^), there are a total of 9 groups of experiments, and each experiment is repeated 30 times. The other parameters in the experiment are consistent with those in Table 4. For a multiobjective GFJSP, the degree of Pareto optimality is used as the response factor, which indicates the percentage of nondominated solutions obtained by the current experiment that are not dominated by the solutions obtained by the other 8 groups of experiments. The experimental results for different combinations of parameters are analyzed by Minitab. The final parameter settings of IMOSSA are displayed in Table 5 for the small, medium, and large cases. The optimal combinations of parameters are *PP* = 0.5, *PD* = 0.1, and *ST* = 0.5 for the small-scale problems; *PP* = 0.5, *PD* = 0.1, and *ST* = 0.5 for the medium-scale problems; and *PP* = 0.5, *PD* = 0.3, and *ST* = 0.65 for the large-scale problems.

**Table 5:** Analysis results of Taguchi’s experiments for IMOSSA parameter settings.

Problem scale	Level	Parameters		
		PP	PD	ST
Small	1	0.530	0.325	0.346
	2	0.082	0.079	0.062
	3	0.006	0.231	0.245
	Range R	0.58	0.24	0.28
	Rank of major factors	1	3	2
	Optimal level	1	1	1
	Optimal combination	0.5	0.1	0.5
Medium	1	0.341	0.353	0.211
	2	0.175	0.184	0.168
	3	0.084	0.073	0.215
	Range R	0.255	0.268	0.057
	Rank of major factors	2	1	3
	Optimal level	1	1	1
	Optimal combination	0.5	0.1	0.5
Large	1	0.143	0.132	0.025
	2	0.053	0.065	0.237
	3	0.141	0.136	0.081
	Range R	0.082	0.068	0.197
	Rank of major factors	2	3	1
	Optimal level	1	3	2
	Optimal combination	0.5	0.3	0.65

As the Jaya algorithm does not require parameterization, the above DOE method is used to test the parameters of NSGA-II and find the optimal ones. There are two important parameters of NSGA-II, including the crossover probability *CP* and mutation probability *MP*, where *CP* ∈ {0.5, 0.55, 0.6} and *MP* ∈ {0.1, 0.15, 0.2}. The optimal combinations of parameters for different problem scales are represented in Table 6.

**Table 6:** Parameter settings of NSGA-II in different-scale cases.

Problem scale	NSGA-II	
	*CP*	*MP*
Small	0.5	0.1
Medium	0.55	0.15
Large	0.6	0.2

### Performance measures

To evaluate the performance of the proposed multiobjective algorithms, we introduce the following measures.

1. Inverted Generational Distance (IGD)^45^: This reflects the average distance between the real Pareto optimal solutions and the Pareto solutions. A lower value of IGD indicates better convergence and implies that the solution set of the algorithm is more approximate and distributed.
  17 $$ IGD = \sqrt {\frac{1}{{\left| {NP} \right|}}\sum\nolimits_{np \in NP} {Dis(np,ps)^{2} } } , $$
  where *NP* represents the real Pareto optimal solution set, *np* is the solution in the set of real Pareto optimal solutions. Since we do not have the existing Pareto optimal solution set for the problem, based on the study of Saber and Ranjbar^45^, we consider all non-dominated solutions found by IMOSSA, NSGA-II, Jaya and CPLEX12.0 as the Pareto-optimal solution set. *ps* is the Pareto solution obtained by the proposed algorithm. Note that *Dis*(*np*, *ps*) is the minimum Euclidian distance between a solution *np* and its nearest solution *ps*.
2. Hypervolume (HV)^46^: This reflects the volume of the nondominated solutions and the selected reference point. Obviously, solutions with higher HV values are better because they dominate a larger area in the objective space.
  18 $$ HV = \zeta \left( { \cup_{pf \in PF} H\left( {pf,r} \right)} \right), $$
  where $\zeta$ represents the Lebesgue measure and $H\left( {pf,r} \right)$ denotes a hypervolume formed between the solutions in the obtained Pareto front *PF* and the selected reference point *r*.

### Results and analysis

We tested the GFJSP using 3 cases of the FT benchmark and 8 cases of the LA benchmark in the OR-Library data. Each case was solved 30 times, and noninferior solutions in terms of makespan and carbon emissions were obtained in each run. The proposed approaches IMOSSA, NSGA-II and Jaya were independently run 30 times for each case. In addition, to verify the effectiveness of IMOSSA, IBM ILOG CPLEX12.0 was used to solve the 11 cases 30 times. A set of non-inferior solutions with respect to makespan (*C*_*max*_), and *TCE* was obtained, and the quality of the non-inferior solutions was evaluated using IGD and HV.

Table 7 reports the performance evaluation results of IMOSSA, NSGA-II, Jaya and CPLEX12.0. After 30 runs, the average values of IGD and HV for each algorithm and CPLEX12.0 were obtained. For simplicity, the optimal values of the three algorithms and CPLEX12.0 in each test case are marked in bold. We can see that IMOSSA obtained better values of the IGD and HV measures, indicated by smaller IGD values and larger HV values. In terms of the IGD measure, IMOSSA was superior to NSGA-II, Jaya and CPLEX12.0 in 8 cases (FT06, FT20, LA01, LA06, LA11, LA16, LA21, LA36). In terms of the HV measure, IMOSSA outperformed NSGA-II, Jaya and CPLEX12.0 in 7 cases (FT10, LA01, LA11, LA16, LA21, LA31, LA36). The evaluation results show that the proposed IMOSSA has better convergence and distribution performance in solving the green flexible job shop scheduling problem.

**Table 7:** Comparison of the performance measure results of three algorithms.

Test cases	$$\begin{array}{*{20}c} n \\ \times \\ m \\ \end{array}$$	IMOSSA		NSGA-II		Jaya		CPLEX12.0	
		IGD	HV	IGD	HV	IGD	HV	IGD	HV
FT06	6 × 6	**0.412**	0.489	2.454	0.428	4.718	**0.503**	0.627	0.348
FT10	10 × 10	0.936	**0.551**	**0.523**	0.534	2.524	0.467	0.641	0.536
FT20	20 × 5	**0.637**	0.524	1.675	0.486	3.846	0.428	0.970	**0.733**
LA01	10 × 5	**1.103**	**0.507**	1.929	0.492	3.063	0.499	1.935	0.479
LA06	15 × 5	**1.315**	0.316	1.842	**0.514**	4.194	0.211	1.652	0.443
LA11	20 × 5	**0.907**	**0.637**	2.064	0.533	3.262	0.497	1.858	0.610
LA16	10 × 10	**0.937**	**0.508**	1.756	0.471	4.145	0.452	1.675	0.497
LA21	15 × 10	**1.393**	**0.634**	2.316	0.630	3.647	0.568	1.436	0.594
LA26	20 × 10	1.348	0.613	**0.958**	0.549	4.813	0.604	1.449	**0.858**
LA31	30 × 10	1.422	**0.691**	1.872	0.684	3.114	0.648	**1.137**	0.673
LA36	15 × 15	**1.534**	**0.775**	3.321	0.724	3.363	0.653	2.419	0.714

Significant values are in bold.

In addition, analysis of variance (ANOVA) was applied to test the significance of the IMOSSA, NSGA-II and Jaya performance under the IGD and HV measures. Table 8 shows the ANOVA test results, where the confidence level is 95%. It is obvious that at different problem scales, the P values of IGD and HV are lower than the significance level of 0.05. This indicates that the values of IGD and HV obtained by IMOSSA are significantly better than those of NSGA-II and Jaya.

**Table 8:** ANOVA table for IGD and HV at different problem scales.

Problem scale		IGD				HV			
		Sum of squares	Degrees of freedom	Mean of squares	P	Sum of squares	Degrees of freedom	Mean of squares	P
Small	Algorithm	0.265	2	0.136	0.002	2.156	2	1.076	0.000
	Error	1.364	8	0.020		3.759	8	0.057	
	Total	1.629	10			5.915	10		
Medium	Algorithm	0.831	2	0.423	0.000	3.951	2	1.971	0.000
	Error	0.796	8	0.013		2.243	8	0.032	
	Total	1.627	10			6.194	10		
Large	Algorithm	1.452	2	0.724		4.921	2	2.463	0.000
	Error	0.534	8	0.006	0.000	0.989	8	0.021	
	Total	1.986	10			5.91	10		

**Figure 7:**
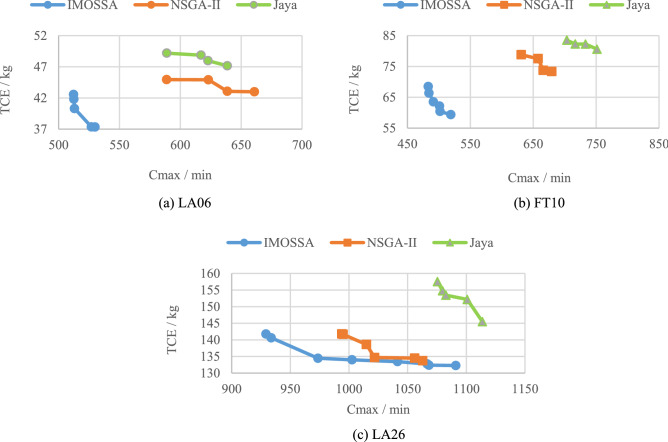
The Pareto fronts of LA06, FT10 and LA26.

To further test the proposed algorithm, we select one test case from each of the three case sizes, small, medium, and large, and plot the Pareto front graphs of the solution sets obtained by the three algorithms. In Fig. 7, the Pareto front obtained by IMOSSA is depicted by the blue points, while those of NSGA-II and Jaya are shown with the orange square points and the green triangular points, respectively. From Fig. 7, we can see that the proposed IMOSSA significantly outperforms the other two comparison algorithms in terms of the quality and quantity of the Pareto optimal points. In addition, for a single machine, there is a possible correlation between its carbon emissions and processing time. However, in the optional machine set, when a machine has the shortest processing time, it is likely to have the highest carbon emissions. For instance, in case LA06, for the optional machine set of job 2, the processing time of machine 5 and machine 3 is 16 min and 26 min respectively, while machine 3 generates more carbon emissions (464 kg) than machine 5 (338 kg). Therefore, two objectives cannot be simultaneously minimized when scheduling, and only Pareto optimal solutions are obtained. Pareto fronts of Fig. 7 verifies the effectiveness of the proposed algorithm.

**Figure 8:**
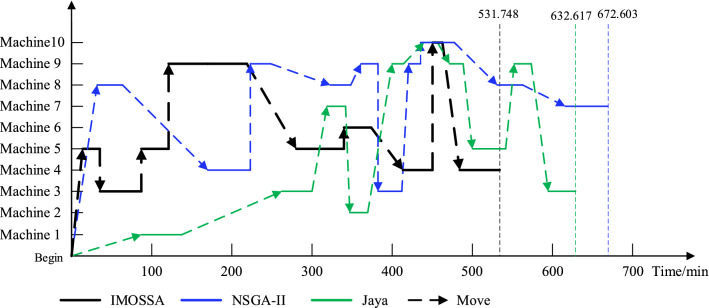
Critical paths in the FT10 case.

Finally, according to Li et al.^47^, the critical path is defined as the longest path from the start of the first process to the completion of the last process, and this determines the final completion time of the entire production process and has a significant impact on the production efficiency. In addition, for the three scheduling schemes in the FT10 case, the critical path of the last finished job is shown in Fig. 8. IMOSSA yields the minimum critical path (makespan); in other words, in regard to solving the GFJSP, IMOSSA has a significant edge over the other two algorithms.

**Figure 9:**
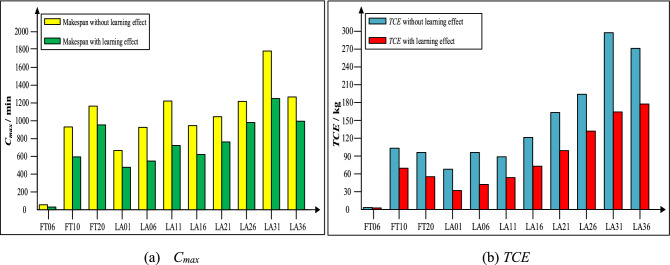
Comparison of *C*_*max*_ and *TCE* with and without the learning effect for 11 cases.

The data shown in Fig. 9 are all obtained by IMOSSA. It is shown that when the learning effect is considered, the percentage reductions in makespan (*C*_*max*_) are 46%, 36%, 28%, 41%, 41%, 34%, 28%, 20%, 30% and 22% for the 11 cases, and the percentage reductions in *TCE* are 29%, 39%, 40%, 28%, 37%, 39%, 40%, 41%, 30%, 41% and 34% for the 11 cases. This indicates that the *C*_*max*_ and *TCE* for the GFJSP are significantly influenced by the learning effect.

## Conclusion

The purpose of this research is to explore the issue of green scheduling in flexible job shop systems while considering the effect of learning. This study focuses on improved formulations and algorithms for solving the GFJSP with multiple objectives related to both economic and environmental concerns. In this study, a mixed integer linear multiobjective optimization model is built to optimize the makespan and carbon emissions. An improved multi-objective sparrow search algorithm (IMOSSA is used to solve the proposed model. Computational experiments are conducted, and the results indicate that IMOSSA has good performance in generating optimal solutions for the GFJSP with a learning effect. Compared with NSGA-II and Jaya in 11 cases, IMOSSA is proven to outperform them.

Future research could continue to concentrate on the GFJSP with a learning effect. Factors in the real manufacturing process, such as time-dependent learning effects, random processing times and job insertion, will be considered in the GFJSP. Additionally, we will explore energy-saving measures in job shop scheduling systems.

## Supplementary Information

Supplementary Information.

## Data Availability

The datasets analyzed during the current study are available in the Appendix. If someone wants to request the data from this study, please contact lizhi@tiangong.edu.cn.
