# Supplementary material for: Minimizing the makespan and carbon emissions in the green flexible job shop scheduling problem with learning effects
Source: Sci Rep. 2023 Apr 19;13:6369. doi: 10.1038/s41598-023-33615-z (PMC10115896; doi:10.1038/s41598-023-33615-z)
Supplement: Supplementary file 1 — Supplementary Information. [file 41598_2023_33615_MOESM1_ESM.docx]

Appendix

FT06:

Processing time = [2 1 0 3 1 6 3 7 5 3 4 6

1 8 2 5 4 10 5 10 0 10 3 4

2 5 3 4 5 8 0 9 1 1 4 7

1 5 0 5 2 5 3 3 4 8 5 9

2 9 1 3 4 5 5 4 0 3 3 1

1 3 3 3 5 9 0 10 4 4 2 1];

Carbon emissions when the machine is running = [22 18 20 19 15 25 ];

Carbon emissions when the machine is idle = [5 4 4 3 1 5 ];

FT10:

Processing time = [0 29 1 78 2 9 3 36 4 49 5 11 6 62 7 56 8 44 9 21

0 43 2 90 4 75 9 11 3 69 1 28 6 46 5 46 7 72 8 30

1 91 0 85 3 39 2 74 8 90 5 10 7 12 6 89 9 45 4 33

1 81 2 95 0 71 4 99 6 9 8 52 7 85 3 98 9 22 5 43

2 14 0 6 1 22 5 61 3 26 4 69 8 21 7 49 9 72 6 53

2 84 1 2 5 52 3 95 8 48 9 72 0 47 6 65 4 6 7 25

1 46 0 37 3 61 2 13 6 32 5 21 9 32 8 89 7 30 4 55

2 31 0 86 1 46 5 74 4 32 6 88 8 19 9 48 7 36 3 79

0 76 1 69 3 76 5 51 2 85 9 11 6 40 7 89 4 26 8 74

1 85 0 13 2 61 6 7 8 64 9 76 5 47 3 52 4 90 7 45];

Carbon emissions when the machine is running = [22 14 21 18 25 30 12 14 20 27];

Carbon emissions when the machine is idle = [2 6 9 4 1 8 10 3 5 2];

FT20:

Processing time = [ 0 29 1 9 2 49 3 62 4 44

0 43 1 75 3 69 2 46 4 72

1 91 0 39 2 90 4 12 3 45

1 81 0 71 4 9 2 85 3 22

2 14 1 22 0 26 3 21 4 72

2 84 1 52 4 48 0 47 3 6

1 46 0 61 2 32 3 32 4 30

2 31 1 46 0 32 3 19 4 36

0 76 3 76 2 85 1 40 4 26

1 85 2 61 0 64 3 47 4 90

1 78 3 36 0 11 4 56 2 21

2 90 0 11 1 28 3 46 4 30

0 85 2 74 1 10 3 89 4 33

2 95 0 99 1 52 3 98 4 43

0 6 1 61 4 69 2 49 3 53

1 2 0 95 3 72 4 65 2 25

0 37 2 13 1 21 3 89 4 55

0 86 1 74 4 88 2 48 3 79

1 69 2 51 0 11 3 89 4 74

0 13 1 7 2 76 3 52 4 45];

Carbon emissions when the machine is running = [14 18 13 23 29];

Carbon emissions when the machine is idle = [4 5 7 10 2];

LA01:

Processing time = [ 1 21 0 53 4 95 3 55 2 34

0 21 3 52 4 16 2 26 1 71

3 39 4 98 1 42 2 31 0 12

1 77 0 55 4 79 2 66 3 77

0 83 3 34 2 64 1 19 4 37

1 54 2 43 4 79 0 92 3 62

3 69 4 77 1 87 2 87 0 93

2 38 0 60 1 41 3 24 4 83

3 17 1 49 4 25 0 44 2 98

4 77 3 79 2 43 1 75 0 96];

Carbon emissions when the machine is running = [14 18 13 23 29];

Carbon emissions when the machine is idle = [4 5 7 10 2];

LA06:

Processing time = [ 1 21 2 34 4 95 0 53 3 55

3 52 4 16 1 71 2 26 0 21

2 31 0 12 1 42 3 39 4 98

3 77 1 77 4 79 0 55 2 66

4 37 3 34 2 64 1 19 0 83

2 43 1 54 0 92 3 62 4 79

0 93 3 69 1 87 4 77 2 87

0 60 1 41 2 38 4 83 3 24

2 98 3 17 4 25 0 44 1 49

0 96 4 77 3 79 1 75 2 43

4 28 2 35 0 95 3 76 1 7

0 61 4 10 2 95 1 9 3 35

4 59 3 16 1 91 2 59 0 46

4 43 1 52 0 28 2 27 3 50

0 87 1 45 2 39 4 9 3 41];

Carbon emissions when the machine is running = [14 18 13 23 29];

Carbon emissions when the machine is idle = [4 5 7 10 2];

LA11:

Processing time = [ 2 34 1 21 0 53 3 55 4 95

0 21 3 52 1 71 4 16 2 26

0 12 1 42 2 31 4 98 3 39

2 66 3 77 4 79 0 55 1 77

0 83 4 37 3 34 1 19 2 64

4 79 2 43 0 92 3 62 1 54

0 93 4 77 2 87 1 87 3 69

4 83 3 24 1 41 2 38 0 60

4 25 1 49 0 44 2 98 3 17

0 96 1 75 2 43 4 77 3 79

0 95 3 76 1 7 4 28 2 35

4 10 2 95 0 61 1 9 3 35

1 91 2 59 4 59 0 46 3 16

2 27 1 52 4 43 0 28 3 50

4 9 0 87 3 41 2 39 1 45

1 54 0 20 4 43 3 14 2 71

4 33 1 28 3 26 0 78 2 37

1 89 0 33 2 8 3 66 4 42

4 84 0 69 2 94 1 74 3 27

4 81 2 45 1 78 3 69 0 96];

Carbon emissions when the machine is running = [14 18 13 23 29];

Carbon emissions when the machine is idle = [4 5 7 10 2];

LA16:

Processing time = [ 1 21 6 71 9 16 8 52 7 26 2 34 0 53 4 21 3 55 5 95

4 55 2 31 5 98 9 79 0 12 7 66 1 42 8 77 6 77 3 39

3 34 2 64 8 62 1 19 4 92 9 79 7 43 6 54 0 83 5 37

1 87 3 69 2 87 7 38 8 24 9 83 6 41 0 93 5 77 4 60

2 98 0 44 5 25 6 75 7 43 1 49 4 96 9 77 3 17 8 79

2 35 3 76 5 28 9 10 4 61 6 9 0 95 8 35 1 7 7 95

3 16 2 59 0 46 1 91 9 43 8 50 6 52 5 59 4 28 7 27

1 45 0 87 3 41 4 20 6 54 9 43 8 14 5 9 2 39 7 71

4 33 2 37 8 66 5 33 3 26 7 8 1 28 6 89 9 42 0 78

8 69 9 81 2 94 4 96 3 27 0 69 7 45 6 78 1 74 5 84];

Carbon emissions when the machine is running = [22 14 21 18 25 30 12 14 20 27];

Carbon emissions when the machine is idle = [2 6 9 4 1 8 10 3 5 2];

LA21:

Processing time = [ 2 34 3 55 5 95 9 16 4 21 6 71 0 53 8 52 1 21 7 26

3 39 2 31 0 12 1 42 9 79 8 77 6 77 5 98 4 55 7 66

1 19 0 83 3 34 4 92 6 54 9 79 8 62 5 37 2 64 7 43

4 60 2 87 8 24 5 77 3 69 7 38 1 87 6 41 9 83 0 93

8 79 9 77 2 98 4 96 3 17 0 44 7 43 6 75 1 49 5 25

8 35 7 95 6 9 9 10 2 35 1 7 5 28 4 61 0 95 3 76

4 28 5 59 3 16 9 43 0 46 8 50 6 52 7 27 2 59 1 91

5 9 4 20 2 39 6 54 1 45 7 71 0 87 3 41 9 43 8 14

1 28 5 33 0 78 3 26 2 37 7 8 8 66 6 89 9 42 4 33

2 94 5 84 6 78 9 81 1 74 3 27 8 69 0 69 7 45 4 96

1 31 4 24 0 20 2 17 9 25 8 81 5 76 3 87 7 32 6 18

5 28 9 97 0 58 4 45 6 76 3 99 2 23 1 72 8 90 7 86

5 27 9 48 8 27 7 62 4 98 6 67 3 48 0 42 1 46 2 17

1 12 8 50 0 80 2 50 9 80 3 19 5 28 6 63 4 94 7 98

4 61 3 55 6 37 5 14 2 50 8 79 1 41 9 72 7 18 0 75];

Carbon emissions when the machine is running = [22 14 21 18 25 30 12 14 20 27];

Carbon emissions when the machine is idle = [2 6 9 4 1 8 10 3 5 2];

LA26:

Processing time = [ 8 52 7 26 6 71 9 16 2 34 1 21 5 95 4 21 0 53 3 55

4 55 5 98 3 39 9 79 0 12 8 77 6 77 7 66 2 31 1 42

5 37 4 92 2 64 6 54 1 19 7 43 0 83 3 34 9 79 8 62

1 87 5 77 0 93 3 69 2 87 7 38 8 24 6 41 9 83 4 60

2 98 5 25 6 75 9 77 1 49 3 17 8 79 0 44 7 43 4 96

1 7 4 61 0 95 2 35 9 10 8 35 5 28 3 76 7 95 6 9

5 59 9 43 0 46 4 28 6 52 3 16 2 59 1 91 8 50 7 27

5 9 9 43 8 14 7 71 4 20 6 54 3 41 0 87 1 45 2 39

1 28 8 66 0 78 2 37 9 42 3 26 5 33 6 89 4 33 7 8

4 96 3 27 6 78 5 84 2 94 8 69 1 74 9 81 7 45 0 69

4 24 7 32 9 25 2 17 3 87 8 81 5 76 6 18 1 31 0 20

8 90 5 28 1 72 7 86 2 23 3 99 6 76 9 97 4 45 0 58

2 17 4 98 3 48 1 46 8 27 6 67 7 62 0 42 9 48 5 27

0 80 8 50 3 19 7 98 5 28 2 50 4 94 6 63 1 12 9 80

9 72 0 75 4 61 8 79 6 37 2 50 5 14 3 55 7 18 1 41

3 96 2 14 5 57 0 47 7 65 4 75 8 79 1 71 6 60 9 22

1 31 7 47 8 58 3 32 4 44 5 58 6 34 0 33 2 69 9 51

1 44 7 40 2 17 0 62 8 66 6 15 3 29 9 38 5 8 4 97

2 58 3 50 4 63 9 87 0 57 6 21 7 57 8 32 1 39 5 20

1 85 0 84 5 56 3 61 9 15 7 70 8 30 2 90 6 67 4 20];

Carbon emissions when the machine is running = [22 14 21 18 25 30 12 14 20 27];

Carbon emissions when the machine is idle = [2 6 9 4 1 8 10 3 5 2];

LA31:

Processing time = [ 4 21 7 26 9 16 2 34 3 55 8 52 5 95 6 71 1 21 0 53

8 77 5 98 1 42 7 66 2 31 3 39 6 77 9 79 4 55 0 12

2 64 4 92 3 34 1 19 8 62 6 54 7 43 0 83 9 79 5 37

0 93 8 24 3 69 7 38 5 77 2 87 4 60 6 41 1 87 9 83

9 77 0 44 4 96 8 79 6 75 2 98 5 25 3 17 7 43 1 49

3 76 2 35 5 28 0 95 7 95 4 61 8 35 1 7 6 9 9 10

1 91 7 27 8 50 3 16 4 28 5 59 6 52 0 46 2 59 9 43

1 45 7 71 2 39 0 87 8 14 6 54 3 41 9 43 5 9 4 20

2 37 3 26 4 33 9 42 0 78 6 89 7 8 8 66 1 28 5 33

1 74 0 69 5 84 3 27 9 81 7 45 8 69 2 94 6 78 4 96

5 76 7 32 6 18 0 20 3 87 2 17 9 25 4 24 1 31 8 81

9 97 8 90 5 28 7 86 0 58 1 72 2 23 6 76 3 99 4 45

9 48 5 27 6 67 7 62 4 98 0 42 1 46 8 27 3 48 2 17

9 80 3 19 5 28 1 12 4 94 6 63 7 98 8 50 0 80 2 50

2 50 1 41 4 61 8 79 5 14 9 72 7 18 3 55 6 37 0 75

9 22 5 57 4 75 2 14 7 65 3 96 1 71 0 47 8 79 6 60

3 32 2 69 4 44 1 31 9 51 0 33 6 34 5 58 7 47 8 58

8 66 7 40 2 17 0 62 9 38 5 8 6 15 3 29 1 44 4 97

3 50 2 58 6 21 4 63 7 57 8 32 5 20 9 87 0 57 1 39

4 20 6 67 1 85 2 90 7 70 0 84 8 30 5 56 3 61 9 15

6 29 0 82 4 18 3 38 7 21 8 50 1 23 5 84 2 45 9 41

3 54 9 37 6 62 5 16 0 52 8 57 4 54 2 38 7 74 1 52

4 79 1 61 8 11 0 81 7 89 6 89 5 57 3 68 9 81 2 30

9 24 1 66 4 32 3 33 8 8 2 20 6 84 0 91 7 55 5 20

3 54 2 64 6 83 9 40 7 8 0 7 4 19 5 56 1 39 8 7

1 6 4 74 0 63 2 64 9 15 6 42 7 98 8 61 5 40 3 91

1 80 3 75 0 26 2 87 9 22 7 39 8 24 4 75 6 44 5 6

5 8 3 79 6 61 1 15 0 12 7 43 8 26 9 22 2 20 4 80

1 36 0 63 7 10 4 22 3 96 5 40 9 5 8 18 6 33 2 62

4 8 8 15 2 64 3 95 1 96 6 38 7 18 9 23 5 64 0 89];

Carbon emissions when the machine is running = [22 14 21 18 25 30 12 14 20 27];

Carbon emissions when the machine is idle = [2 6 9 4 1 8 10 3 5 2];

LA36:

Processing time = [ 4 21 3 55 6 71 14 98 10 12 2 34 9 16 1 21 0 53 7 26 8 52 5 95 12 31 11 42 13 39

11 54 4 83 1 77 7 64 8 34 14 79 12 43 0 55 3 77 6 19 9 37 5 79 10 92 13 62 2 66

9 83 5 77 2 87 7 38 4 60 12 98 0 93 13 17 6 41 10 44 3 69 11 49 8 24 1 87 14 25

5 77 0 96 9 28 6 7 4 95 13 35 7 35 8 76 11 9 12 95 2 43 1 75 10 61 14 10 3 79

10 87 4 28 8 50 2 59 0 46 11 45 14 9 9 43 6 52 7 27 1 91 13 41 3 16 5 59 12 39

0 20 2 71 4 78 13 66 3 14 12 8 14 42 6 28 1 54 9 33 11 89 8 26 7 37 10 33 5 43

8 69 4 96 12 17 0 69 7 45 11 31 6 78 10 20 3 27 13 87 1 74 5 84 14 76 2 94 9 81

4 58 13 90 11 76 3 81 7 23 9 28 1 18 2 32 12 86 8 99 14 97 0 24 10 45 6 72 5 25

5 27 1 46 6 67 8 27 13 19 10 80 2 17 3 48 7 62 11 12 14 28 4 98 0 42 9 48 12 50

11 37 5 80 4 75 8 55 7 50 0 94 9 14 6 41 14 72 3 50 10 61 13 79 2 98 12 18 1 63

7 65 3 96 0 47 4 75 12 69 14 58 10 33 1 71 9 22 13 32 5 57 8 79 2 14 11 31 6 60

1 34 2 47 3 58 5 51 4 62 6 44 9 8 7 17 10 97 8 29 11 15 13 66 12 40 0 44 14 38

3 50 7 57 13 61 5 20 11 85 12 90 2 58 4 63 10 84 1 39 9 87 6 21 14 56 8 32 0 57

9 84 7 45 5 15 14 41 10 18 4 82 11 29 2 70 1 67 3 30 13 50 6 23 0 20 12 21 8 38

9 37 10 81 11 61 14 57 8 57 0 52 7 74 6 62 12 30 1 52 2 38 13 68 4 54 3 54 5 16 ];

Carbon emissions when the machine is running = [16 10 14 29 13 29 23 18 26 29 15 24 28 15 27];

Carbon emissions when the machine is idle = [6 10 2 5 7 6 10 7 3 4 5 6 8 1 5].
